# Three-dimensional quantitative temporomandibular joint changes in skeletal class I malocclusion treated with extraction and non-extraction protocols: a comparative study of fixed orthodontic appliances and clear aligners

**DOI:** 10.1186/s40510-024-00551-1

**Published:** 2025-01-20

**Authors:** Majedh Abdo Ali Al-Somairi, Bowen Zheng, Xaiofeng Yang, Yongxin Zhang, Maged S. Alhammadi, Hao xu, Najah Alhashimi, Bushra Sufyan Almaqrami, Naseem Ali Al –Worafi, Enas Senan Alyafrusee, Barakat Al-Tayar, Yi Liu

**Affiliations:** 1 Department of Orthodontics, School and Hospital of Stomatology, China Medical University, Shenyang, 110002 P.R. China; 2 Department of Orthodontics and Dentofacial Orthopedics, Ibb University, Ibb, Yemen; 3 Department of Preventive Dental Sciences, College of Dentistry, Jazan University, Jazan, Saudi Arabia; 4 Unit and Division Chief Orthodontics at Hamad Medical Corporation, and associate professor, College of Dental Medicine, Qatar University, Doha, Qatar; 5 Orthodontics Division, Faculty of Medicine and Health Sciences, Taiz University, Taiz, Yemen; 6 Department of Orthodontics, Ningbo Dental Hospital, Ningbo, China

**Keywords:** Temporomandibular joint, Cone beam computed tomography, Fixed appliances, Clear aligners, Extraction, Non-extraction, Malocclusion

## Abstract

**Objective:**

This study aimed to evaluate the positional and morphological changes in the temporomandibular joint (TMJ) in adult patients with skeletal Class I malocclusion treated with fixed orthodontic appliances (FAs) and clear aligners (CAs), both with and without premolar extractions.

**Methods:**

This retrospective study involved 120 adult patients divided into non-extraction and extraction groups, each further subdivided equally into those treated with FAs and CAs. Cone beam computed tomography (CBCT) was used to assess the TMJ measurements before (T0) and after treatment (T1). Statistical analyses were conducted to compare the mean changes in intra- and inter-groups. A significance level of *p* ≤ 0.05 was considered.

**Results:**

In the non-extraction group, specifically in FAs, significant increases were observed in TMJ parameters; anteroposterior condylar position (APCP) and mediolateral condylar inclination (MCI). Conversely, significant decreases were noted in vertical condylar position (VCP) and vertical condylar inclination (VCI). In the extraction group, significant increases were noted in APCP and anterior joint space (AJS), while posterior joint space (PJS) and anteroposterior condylar joint position (APCJP) decreased. For inter-group comparisons, the extraction group showed significant increases in APCP in FAs compared to CAs, and a significant decrease in APCJP in FAs compared to CAs.

**Conclusion:**

FAs significantly impact condylar positions and joint spaces, especially in extraction cases. Monitoring TMJ parameters during orthodontic treatment is crucial to ensure positive outcomes and prevent TMJ disorders (TMDs). These findings may guide the selection of orthodontic appliances based on individual malocclusion characteristics.

**Supplementary Information:**

The online version contains supplementary material available at 10.1186/s40510-024-00551-1.

## Introduction

The temporomandibular joint (TMJ) is a complex joint facilitating mandibular movement and adapting structurally in response to external factors such as age, muscle activity, and occlusal forces [1–3]. Malocclusions, such as crossbites, crowding, and missing teeth, are known to influence TMJ morphology and function. However, the relationship between malocclusion and TMJ remains unclear, with some studies showing associations [4–6], while others do not [7]. Most clinical evidence indicates that no direct causal relationship between orthodontic treatment and temporomandibular joint disorders (TMDs) [8]. The relationship between dental occlusion and TMD is debated, with systematic reviews finding no definitive evidence linking occlusal features to TMDs [9]. Orthodontic treatment aims to restore balanced occlusion, which may impact TMJ position and stability [10]. However, evidence regarding their role in TMD development remains inconclusive. Some studies suggest orthodontic interventions may promote TMJ remodeling and improve condyle-glenoid fossa relationship, enhancing joint function [11]. Other researches suggested that orthodontic appliances may disrupt occlusal stability, potentially triggering or worsening TMDs [12, 13]. These findings underscore the need for rigorous research to clarify the complex link between orthodontic treatments, occlusal dynamics, and TMDs.

The orthodontic community is similarly divided on the impact of extractions, with proponents citing benefits for crowding and vertical dimension control [14–16], while opponents argue that extractions may contribute to TMJ dysfunction [17, 18]. Traditional fixed orthodontic appliances (FAs), have long been the standard in orthodontic treatment, providing robust control over tooth movement, Whereas clear aligners (CAs) have emerged as a popular alternative due to their aesthetic advantages and less intrusive impact on TMJ dynamics [19]. Unlike FAs, CAs offer complete tooth crown coverage and precise force application through digitally designed attachments, potentially enhancing control of three-dimensional tooth movement. The aligner thickness at the occlusal surface can act as a 'bite-block,’ aiding vertical dimension control [20]. Despite these benefits, controlling vertical tooth movement remains challenging in orthodontics. Precise movement control is crucial to avoid complications like mandibular rotation [21]. While CAs offer better oral hygiene and reduced enamel demineralization risk, their effectiveness in controlling specific tooth movements is still debated [22].

While cone beam computed tomography (CBCT) has enhanced our ability to assess TMJ structures in three-dimensions (3D) that surpasses conventional methods [23, 24], limited research has focused on evaluating TMJ positional and morphological changes in adults pre- and post-treatment with FAs and CAs, particularly in extraction and non-extraction cases [25, 26]. To the best of the authors’ knowledge, this study is the first to use 3D CBCT to comprehensively compare TMJ structural changes between these approaches. It aims to clarify the impact of FAs and CAs on TMJ stability and remodeling, building on prior research that has highlighted both the potential benefits and risks of orthodontic treatments for TMJ health.

This study aimed to address gaps in the literature by providing new insights into the effects of FAs and CAs on TMJ adaptations through three-dimensional evaluation of TMJ structural changes following treatment with FAs and CAs in both extraction and non-extraction cases, it contributes to refining orthodontic treatment planning and appliance selection for optimal TMJ outcome.

## Materials and methods

### Sample selection

The Ethics Committee of the China Medical University School of Stomatology approved this study (Ethics Approval No. CMUKQ-2024-019), and informed consent was obtained from all participants. All methods were carried out in accordance with the principles of the declaration of Helsinki. This research involved a retrospective review of adult patients’ records treated with FAs or CAs who underwent non-extraction and extraction treatments between 2017 and 2024.

**Fig. 1:**
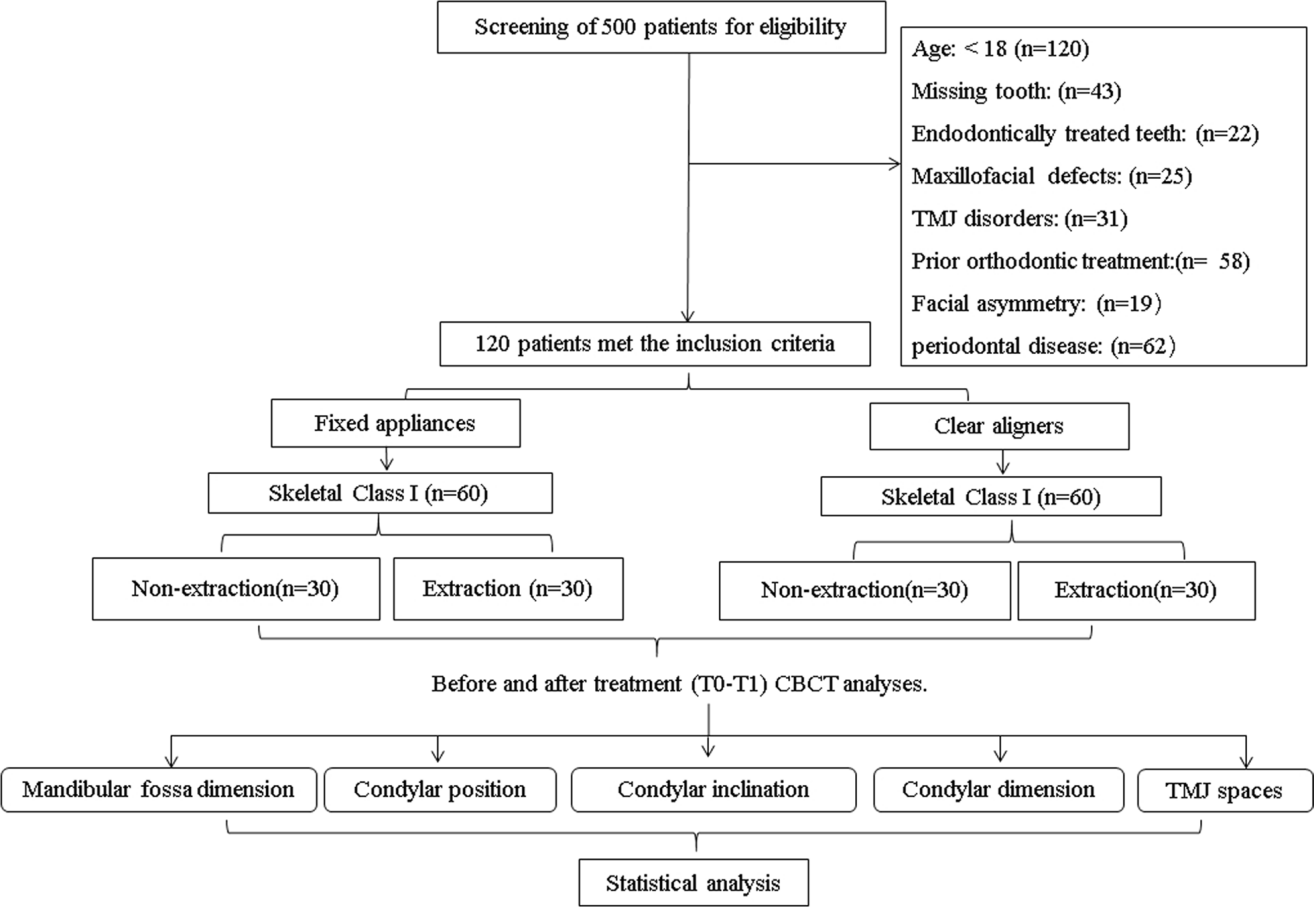
Flow chart of patient selection, outlining inclusion and exclusion criteria leading to the final study cohort

The patient selection process adhered to strict inclusion and exclusion criteria to ensure uniformity across groups. From an initial pool of 500 patients, 120 adult cases were selected after applying the criteria. Participants were categorized into four equal groups (n = 30 per group) based on the treatment modality (FAs or CAs) and treatment protocol (extraction or non-extraction) to allow for direct comparisons of TMJ adaptations under these different clinical scenarios in adults (non-growing patients). All patients meeting the following selection criteria were included: (1) age over 18 years; (2) skeletal Class I malocclusion with moderate crowding in both arches; (3) no missing teeth (second molar to second molar) for the non-extraction group, and four first premolars extracted for the extraction group; (4) no history of TMD symptoms was reported, and TMD diagnoses, based on the DC/TMD, were derived from dental records by assessing TMJ pain, sounds, and mandibular range of motion [27]; and (5) complete treatment records with high-quality pre- and post-treatment CBCT images. Exclusion criteria were: (1) previous orthodontic, prosthodontic, or orthognathic treatment; (2) presence of impacted, supernumerary, or missing teeth; (3) facial asymmetry or functional mandibular deviations; (4) craniofacial syndromes; and (5) periodontal disease. Figure 1 provides a flowchart detailing the patient selection and allocation process, illustrating screening, exclusions, and final group distributions. This flowchart details the screening process, exclusions, and final group distribution, ensuring transparency and reproducibility.

Sample size estimation was calculated using G*Power 3.1, considering a 5% significance level and 95% power, based on the SN-MP angle differences reported by Wang et al. [28]; a sample of 27 patients per group was needed, but this was subsequently increased to 30 for robustness, examining a total of 240 patients data. Each group had 60 patients divided equally between premolars extraction and non-extraction treatment.

In the study, non-extraction patients in the FAs group received treatment with the Damon Q self-locking bracket system from Ormco, US. Treatment started with 0.014-inch or 0.016-inch NiTi wires, progressing to 0.016 × 0.022-inch NiTi wires, and then to stainless steel of the same dimension. After alignment and leveling, molar distalization was achieved using Class I elastics (1/4-in, 4.5 oz, Ormco Corp.) attached from mini-screws to hooks between the lateral incisors and canines [29]. Mini-screws (2 mm diameter, 10 or 12 mm length; Bioray, Taiwan) were placed in the infrazygomatic crest area between the maxillary first and second molars, and in the buccal shelf area between the mandibular first and second molars [41]. Class II elastics were also used to exert a distalization force of about 300 g [28].

In the CAs group, comprehensive orthodontic treatment using Clear Aligners (Align Technology) was applied to all teeth, including second molars. Treatment involved custom attachments per the manufacturer's and orthodontist's specifications. Align Technology's protocol followed a staged approach for sequential molar distalization, with each aligner moving teeth by 0.25 mm. Molar distalization was enhanced by elastics (1/4-inch, 4.5 oz, Ormco Corp.) attached from canines to mini-screws. After distalization, a refinement period was necessary. Patients wore each aligner for 22 h daily for 7–10 days, progressing to the next aligner after a six-week evaluation.

In the extraction groups, extraction space was used to alleviate crowding and retract incisors in both arches. Both FAs and CAs treatments involved three phases: alignment and leveling, space closure, and fine-tuning or finshing. For the FAs group, mini-screws retracted incisors, with archwire adjustments and intermaxillary elastics used for space closure. Treatment concluded once the teeth were properly aligned and all spaces were closed.

For CAs treatments, a standardized protocol was used with attachments on canines, second premolars, and molars for alignment. The process began with the retraction of canines, followed by incisors, repeating as needed to close extraction spaces. Class I, II, and vertical elastics supported space closure and bite adjustments. Patients wore aligners for at least 22 h daily, switching them bi-weekly. Typically, two refinement phases corrected bite openings or completed space closure. Class II elastics ensured dental anchorage, prevented anterior tooth flaring, and enhanced intercuspation [30].

All treatments were conducted by a highly experienced orthodontist, ensuring reliable execution. Factors such as malocclusion severity, biomechanics, extent of tooth movement (extraction and non-extraction), and treatment outcome quality were considered to assess and compare difficulty levels between patient groups. The American Board of Orthodontics (ABO) discrepancy index was used to evaluate case difficulty [31], providing a standardized measure of treatment challenges across all studied groups.

### CBCT analysis

CBCT analysis was conducted using the iCAT CBCT System (KaVo 3D eXam, KaVo Dental, Germany) with a 23 × 17 cm field of view (FOV), 37.1 MAs exposure, 17.8 s scan duration, and 120 kV settings. Images had a 0.3 mm slice thickness and voxel size, with a resolution of 768 × 768 pixels. To minimize motion artifacts, patients maintained the Frankfort Horizontal Plane (FHP) parallel to the floor and were instructed to remain still during the scanning process. The imaging settings were optimized to balance resolution and radiation exposure. The FOV ensured comprehensive bilateral TMJ coverage, while a voxel size of 0.3 mm provided adequate spatial resolution for precise TMJ landmarks identification, as recommended in standard protocols [32].

Pre- and post-treatment CBCT scans were converted into DICOM format and analyzed using Invivo 6.0 software (Anatomage, San Jose, CA, USA).

**Fig. 2:**
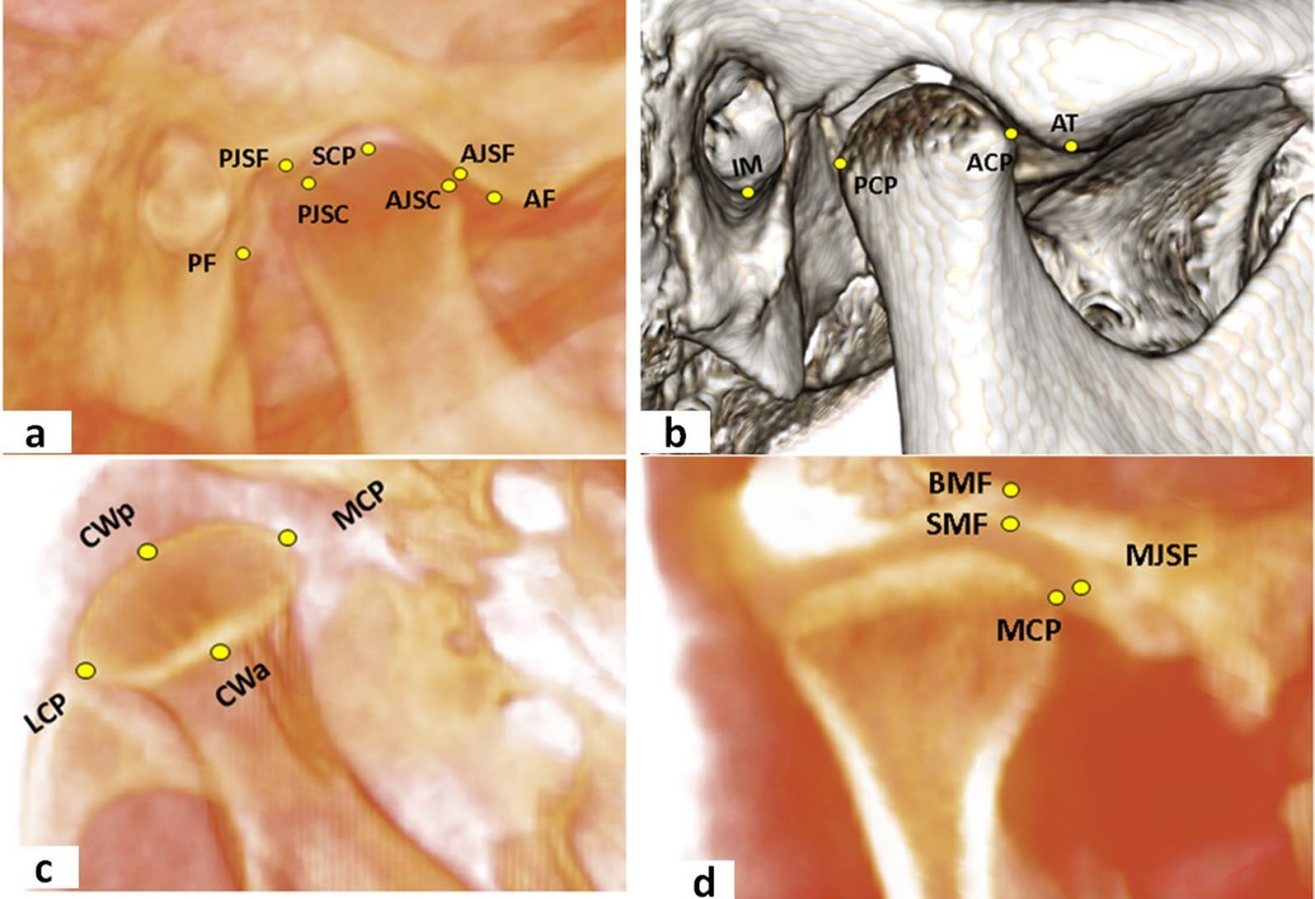
TMJ anatomical landmarks for the CBCT analysis: **a**, **b** sagittal views, **c** axial view, **d** coronal view (landmarks are defined in  supplementary material 1)

**Fig. 3:**
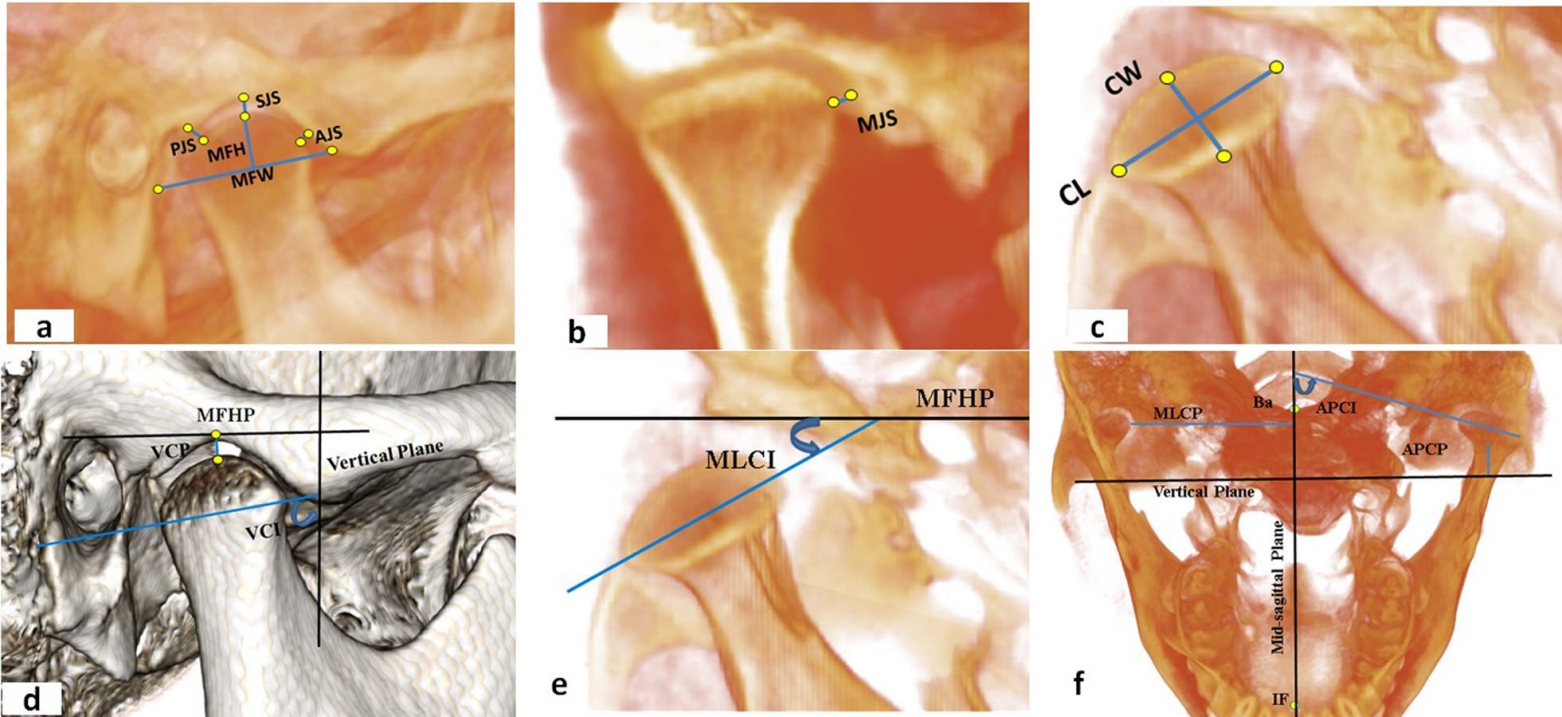
TMJ measurements of the CBCT analysis: **a**, **b** mandibular fossa dimensions (MH and MW) and TMJ spaces (AJS, SJS, PJS, and MJS), **c** condylar dimensions (CL and CW), and **d**, **e**, **f** condylar positions (MLCP, VCP, and APCP) and inclinations (VCI, MLCI, and APCI) (Measurements are defined in supplementary material 3)

The three-dimensional TMJ analysis methodology adopted in our study was based on the protocol established by Alhammadi et al. [33]. This included standardized identification of skeletal and TMJ landmarks, outlined in supplementary material 1 (Fig. 2), with corresponding reference planes and lines presented in supplementary material 2 and the measurements detailed in supplementary material 3 (Fig. 3).

The accuracy and precision of the condylar position were assessed using two methods. First, the condylar position relative to basal craniofacial structures was evaluated using reference planes (MSP, HP, and VP). Second, the formula by Pullinger et al. [34] ((P − A)/(P + A) × 100%) determined the condyle's centrality on the sagittal slice. Condyle positions were categorized using the Pullinger formula as posterior (< − 12%), anterior (> + 12%), or concentric (± 12%), which helps assess joint stability and remodeling during treatment.

**Fig. 4:**
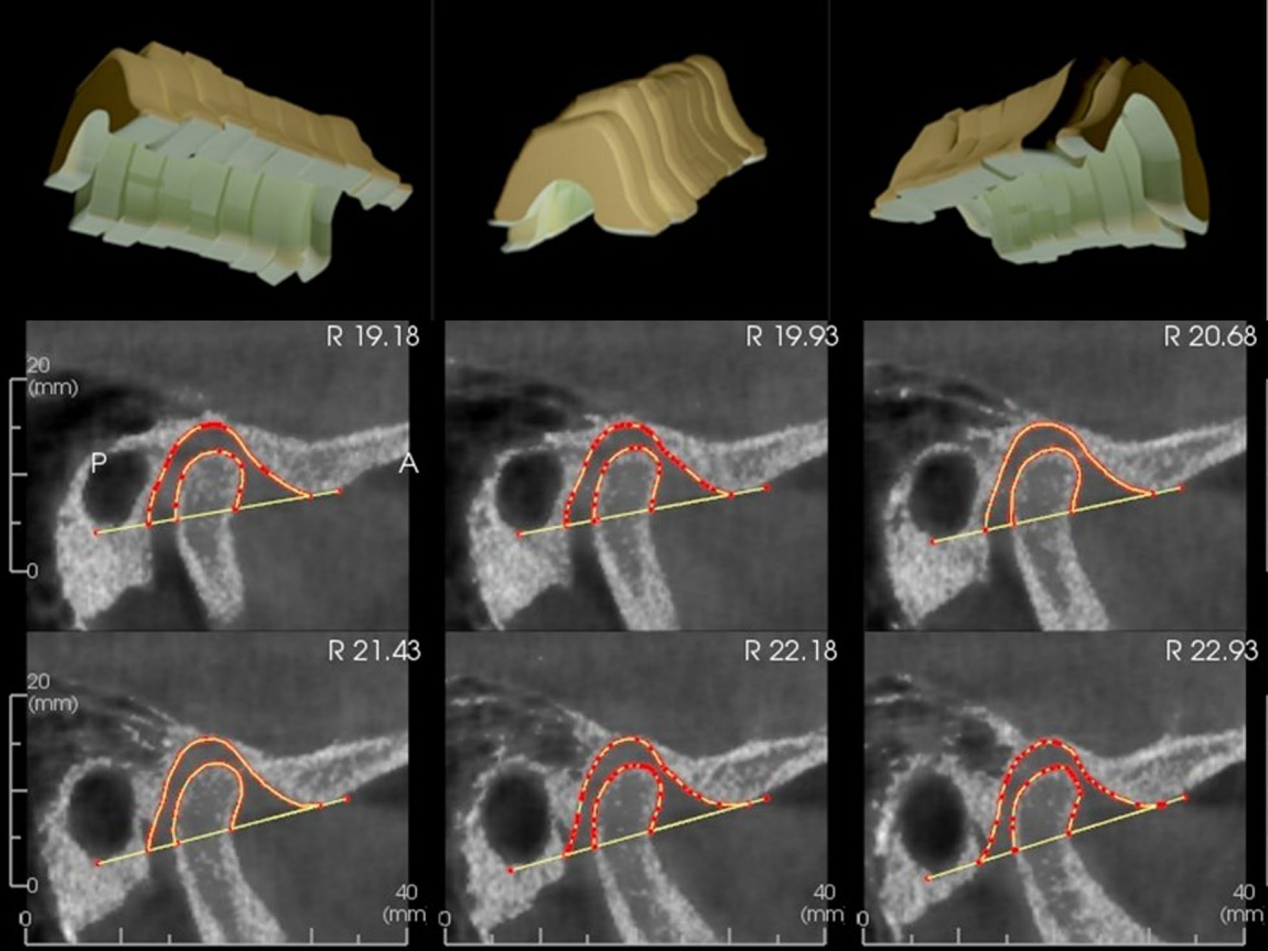
3D representation of total TMJ space volumes with overlaid 2D identification points

Subsequent CBCT images were taken with a 20 × 25 cm field of view, at 110 kV and 8.8 mAs, with an 18-s exposure time. The voxel dimension was 0.3 mm, and the slice thickness was 2 mm, ensuring FHP alignment with a laser guide. TMJ space was segmented into six 1.5 mm sections per side, and volumes were calculated using the sigma volume equation v ≅ Σ_k = 1_ A (x ˙_I_)Δx (Fig. 4) [35].

Intra- and inter-observer reliability were evaluated by randomly selecting 24 CBCT images, which were measured independently one month later to confirm consistency. Measurements were conducted twice within a two-week interval by two observers to ensure reliability.

### Statistical analysis

Data analysis was conducted using SPSS software, version 26 (IBM Corp., Armonk, NY, USA). To ensure the reliability and accuracy of measurements, Intra-class Correlation Coefficients (ICCs) were calculated, and Technical Error of Measurement (TEM) along with Relative Technical Error of Measurement (rTEM) were assessed. Descriptive statistics were used to summarize the data, with continuous variables reported as means and standard deviation (SD), and categorical variables presented as frequencies and percentages. The Shapiro–Wilk test was employed to assess the normality of the data. For statistical comparisons, paired t-tests were used to evaluate intra-group comparisons, while independent t-tests were applied for inter-group comparisons. Baseline differences between groups were assessed using independent *t* tests for continuous variables and Chi-squared tests for categorical variables. Statistical significance was set at p ≤ 0.05 and effect size was measured using Cohen's d when significant results were observed.

## Results

### Sample and descriptive data

In the non-extraction group, 30 patients received FAs (mean age: 22.21 ± 5.20 years) and 30 received CAs (mean age: 24.27 ± 4.27 years). In the extraction group, 30 patients were treated with FAs (mean age: 23.29 ± 4.21 years) and 30 with CAs (mean age: 24.35 ± 4.68 years). No significant differences were found in treatment duration between groups; non-extraction treatments averaged 2.47 ± 0.73 years for FAs and 2.21 ± 0.74 years for CAs (*p* = 0.168), while extraction treatments lasted 3.27 ± 0.85 years with FAs and 2.95 ± 0.94 years with CAs (*p* = 0.173).

No significant differences were found in baseline characteristics, including age, gender, treatment duration, skeletal and dental characteristics, and ABO discrepancy index scores (*p* ≥ 0.05) (Table 1). All patients presented with skeletal Class I malocclusion, moderate crowding, and asymptomatic TMJs at baseline, as determined by clinical and radiological evaluation. The intra- and inter-observer reliability analyses of all measurements showed high reliability using 20% of the total sample (Supplementary material 4).

**Table 1:** Evaluation of pretreatment baseline characteristics and demographic data used in the study

Measurements	Non-extraction				*P*-value	Extraction				*P*-value
	FAs		CAs			FAs		CAs		
	Mean	SD	Mean	SD		Mean	SD	Mean	SD	
Age^+^	22.21	5.20	24.27	4.27	0.082	23.29	4.21	24.35	4.68	0.359
Treatment duration^+^	2.47	0.73	2.21	0.74	0.168	3.27	0.85	2.95	0.94	0.173
Gender (Male/female N (%))*	14(46.7)/16(53.3)		13(43.3)/17(56.7)		0.795	13(43.33)/17(56.6)		12(40.0)/18(30)		0.793
IMP (°)^+^	100.52	7.70	102.12	5.95	0.372	101.93	4.88	102.23	4.33	0.803
ANB (°)^+^	2.72	0.90	2.93	1.05	0.401	2.97	0.86	3.14	0.93	0.468
MP-SN (°)^+^	30.66	2.66	31.7	2.72	0.140	32.31	3.01	30.89	1.92	0.154
Overbite (mm)^+^	2.76	1.31	2.79	1.45	0.472	2.82	0.98	2.99	0.57	0.438
Overjet (mm)^+^	3.63	0.84	4.05	0.98	0.098	4.30	0.97	4.71	0.67	0.322
IMP*	1.50	0.51	1.7	0.47	0.114	1.67	0.48	1.73	0.45	0.770
Overbite*	0.70	0.95	0.87	1.01	0.513	0.87	1.01	1.13	1.01	0.302
Overjet*	1.60	0.93	2.00	0.91	0.098	1.97	0.76	2.20	0.76	0.241
Anterior open bite*	0.30	0.84	0.13	0.43	0.503	0.2	0.41	0.13	0.43	0.204
Lateral open bite*	0.33	0.88	0.20	0.61	0.330	0.33	0.76	0.40	0.97	0.574
Crowding upper*	2.27	0.69	2.4	0.81	0.488	2.67	0.96	3.00	1.02	0.190
Crowding lower*	2.27	0.69	2.47	0.86	0.317	2.80	1.0	3.13	1.01	0.196
Buccal posterior crossbite*	0.47	0.86	0.13	0.51	0.071	0.20	0.61	0.13	0.51	0.640
Lingual posterior crossbite*	0.07	0.25	0.13	0.43	0.601	0.13	0.35	0.17	0.46	0.565
Total score*	8.67	3.03	8.33	2.8	0.348	9.17	2.90	10.30	2.98	0.330

- SD, standard deviation; N, number; ° (degree); %, ratio measurements; mm, millimetres;. + , independent t test; *, Pearson chi-square test

### Intra-group comparisons

Tables 2 and 3 compare TMJ measurements at two time points (T0 and T1) within the non-extraction and extraction groups (FAs and CAs), respectively.

**Table 2:** Intra-group comparison of TMJ parameters (T0-T1) for FAs and CAs in the non-extraction group

Measurements	FAs				*P-*value	CAs				*P-*value
	T0		T1			T0		T1		
	Mean	SD	Mean	SD		Mean	SD	Mean	SD	
*Jaw relation *(°)										
ANB	2.72	0.90	2.67	1.03	0.612	2.92	1.07	2.96	1.00	0.800
MP-SN	30.66	2.66	31.55	2.85	0.000*	31.71	2.77	31.97	2.61	0.269
*Mandibular fossa dimension (mm)*										
MFH	12.07	0.99	12.17	1.13	0.325	11.67	1.44	11.56	2.64	0.753
MFW	17.52	1.49	17.57	1.46	0.300	17.47	1.67	17.51	1.63	0.603
*Condylar dimension (mm)*										
CL	18.52	1.75	18.68	1.85	0.072	19.09	1.52	19.15	1.47	0.607
CW	7.40	0.77	7.54	0.78	0.242	8.05	0.88	8.17	1.19	0.233
*Condylar position (mm)*										
APCP	6.11	1.74	6.49	1.88	0.000*	7.17	2.37	7.32	2.42	0.532
VCP	2.32	1.28	2.13	1.25	0.025*	2.36	1.56	2.27	1.36	0.452
MLCP	44.08	2.10	44.12	2.17	0.769	44.38	2.40	44.48	2.58	0.215
APCJP	10.36	12.20	7.07	14.45	0.093	6.11	15.04	4.84	16.59	0.596
*Condylar inclination* ( ° )										
ACPI	71.77	4.49	72.67	4.74	0.059	70.74	4.83	70.79	4.30	0.914
VCI	58.15	6.53	56.16	6.20	0.022*	58.83	7.16	58.18	6.65	0.423
MCI	8.90	4.80	10.07	4.53	0.034*	10.29	4.47	10.61	5.17	0.629
*TMJ spaces (mm)*										
AJS	2.41	0.50	2.52	0.56	0.166	2.55	0.69	2.60	0.76	0.504
SJS	3.33	0.64	3.21	0.61	0.147	3.91	0.94	3.77	0.97	0.384
PJS	2.98	0.69	2.92	0.64	0.450	2.88	0.56	2.77	0.46	0.150
MJS	2.78	0.53	2.81	0.52	0.726	3.29	0.83	3.34	0.92	0.712
TMJV( mm^3^)	292.50	34.35	288.40	38.77	0.309	290.79	44.56	292.94	48.44	0.685

SD = standard deviation, T0 = before treatment, T1 = after treatment, changes in TMJ parameters (T0 vs. T1) for non-extraction groups, analyzed using paired t-tests. *p ≤ 0.05 indicates statistical significance

**Table 3:** Intra-group comparison of TMJ parameters (T0-T1) for FAs and CAs in the extraction group

Measurements	FAs				*P-*value	CAs				*P-*value
	T0		T1			T0		T1		
	Mean	SD	Mean	SD		Mean	SD	Mean	SD	
*Jaw relation* (°)										
ANB	2.97	0.86	2.41	0.96	0.000*	3.14	0.93	2.81	0.85	0.000*
MP-SN	32.42	2.73	33.83	3.11	0.000*	30.06	1.90	30.24	2.35	0.339
*Mandibular fossa dimension (mm)*										
MFH	11.69	1.03	11.77	1.06	0.218	11.54	1.21	11.65	1.33	0.258
MFW	16.58	1.47	16.65	1.43	0.127	17.99	1.67	18.06	1.68	0.348
*Condylar dimension (mm)*										
CL	17.97	1.95	18.02	2.02	0.749	17.87	0.96	17.86	0.86	0.884
CW	7.49	0.71	7.53	0.60	0.723	8.07	1.23	8.18	1.21	0.086
*Condylar position (mm)*										
APCP	6.58	1.98	7.08	1.84	0.000*	7.86	2.06	8.01	2.23	0.071
VCP	1.98	1.32	1.85	1.48	0.193	2.35	0.94	2.26	1.07	0.087
MLCP	43.22	1.82	43.28	1.75	0.258	43.06	2.22	43.13	2.27	0.272
APCJP	2.39	16.62	-11.78	16.32	0.000*	1.89	16.20	-0.98	13.56	0.152
*Condylar inclination* ( ° )										
ACPI	70.77	5.79	71.16	6.37	0.213	71.50	4.57	72.02	4.25	0.111
VCI	57.20	8.78	56.50	9.61	0.497	63.96	6.45	63.65	8.32	0.714
MCI	7.37	4.97	7.93	4.11	0.380	8.26	4.35	8.92	3.76	0.201
*TMJ spaces (mm)*										
AJS	2.57	0.56	2.85	0.67	0.027*	2.57	0.63	2.71	0.59	0.082
SJS	3.40	0.61	3.31	0.48	0.469	3.66	0.58	3.59	0.59	0.271
PJS	2.68	0.56	2.36	0.47	0.021*	2.68	0.37	2.61	0.33	0.165
MJS	3.15	0.75	3.10	1.04	0.716	2.91	0.53	2.85	0.66	0.523
TMJV( mm^3^)	280.04	42.29	278.88	46.96	0.727	268.74	36.14	265.47	39.44	0.356

SD = standard deviation, T0 = before treatment, T1 = after treatment, changes in TMJ parameters (T0 vs. T1) for extraction groups, analyzed using paired t-tests. *p ≤ 0.05 indicates statistical significance

In the non-extraction FAs group, Table 2, the anteroposterior condylar position increased significantly (from 6.11 to 6.49 mm, *p* = 0.000, effect size = 0.210). The vertical condylar position decreased significantly (from 2.32 to 2.13 mm, *p* = 0.025, effect size = 0.150). The vertical condylar inclination decreased (from 58.15 to 56.16°, *p* = 0.022, effect size = 0.312). The medial condylar inclination increased significantly (from 8.90 to 10.07°, *p* = 0.034, effect size = 0.251).

In the extraction FAs group, Table 3, significant changes were noted, where the anteroposterior condylar position increased significantly (from 6.58 to 7.08 mm, *p* = 0.000, effect size = 0.261). The anteroposterior condylar joint position demonstrated a significant decrease (from 2.39 to − 11.78 mm, *p* = 0.000, effect size = 0.861), and the anterior joint space increased significantly (from 2.57 to 2.85 mm, *p* = 0.027, effect size = 0.454). In contrast, the posterior joint space decreased significantly (from 2.68 to 2.36 mm, *p* = 0.021, effect size = 0.619).

### Inter-group comparisons

Table 4 presents an inter-group comparisons of TMJ measurements (T0-T1) for both non-extraction and extraction groups, highlighting significant differences between the FAs and CAs groups. Negative mean difference values (T0 minus T1) indicate an increase in the respective measurements post-treatment. In the extraction group, FAs demonstrated significant increases in anteroposterior condylar position (*p* = 0.014, effect size = 0.657) and anteroposterior condylar joint position (*p* = 0.046, effect size = 0. 0.525), indicating greater condylar remodeling compared to CAs.

**Table 4:** Inter-group comparison of TMJ parameters (T0-T1) for FAs and CAs within non-extraction and extraction groups

Measurements	Non-extraction				*P-*value	Extraction				*P-*value
	FAs		CAs			FAs		CAs		
	Mean	SD	Mean	SD		Mean	SD	Mean	SD	
*Jaw relation* (°)										
ANB	0.04	0.45	– 0.04	0.83	0.661	0.56	0.56	0.33	0.34	0.065
MP-SN	– 0.97	0.94	– 0.27	1.23	0.017*	– 1.42	1.94	– 0.23	0.97	0.004*
*Mandibular fossa dimension (mm)*										
MFH	– 0.10	0.54	0.10	1.80	0.554	– 0.08	0.33	– 0.12	0.55	0.732
MFW	– 0.06	0.29	– 0.05	0.50	0.939	– 0.06	0.23	– 0.07	0.41	0.923
*Condylar dimension (mm)*										
CL	– 0.16	0.47	– 0.07	0.70	0.536	– 0.05	0.80	0.01	0.53	0.725
CW	– 0.14	0.64	– 0.11	0.50	0.852	– 0.04	0.63	– 0.11	0.34	0.606
*Condylar position (mm)*										
APCP	– 0.37	0.50	– 0.15	1.28	0.372	– 0.50	0.65	– 0.14	0.42	0.014*
VCP	0.19	0.44	0.08	0.73	0.563	0.13	0.54	0.09	0.29	0.739
MLCP	– 0.04	0.72	– 0.10	0.44	0.681	– 0.07	0.31	– 0.06	0.30	0.972
APCJP	3.29	10.38	1.27	12.96	0.509	11.23	19.81	2.87	10.66	0.046*
*Condylar inclination* ( ° )										
ACPI	– 0.91	2.52	– 0.05	2.56	0.198	– 0.39	1.68	– 0.47	1.72	0.861
VCI	1.99	4.50	0.65	4.88	0.292	0.70	5.60	0.06	4.51	0.624
MCI	– 1.17	2.88	– 0.32	3.61	0.320	– 0.56	3.43	– 0.67	2.79	0.894
*TMJ spaces (mm)*										
AJS	– 0.11	0.42	– 0.05	0.39	0.565	– 0.28	0.65	– 0.14	0.43	0.357
SJS	0.12	0.43	0.14	0.88	0.890	0.09	0.66	0.07	0.33	0.877
PJS	0.06	0.42	0.12	0.42	0.588	0.33	0.73	0.07	0.28	0.082
MJS	– 0.03	0.46	– 0.05	0.71	0.906	0.05	0.77	0.06	0.53	0.947
TMJV( mm^3^)	4.10	21.70	– 2.15	28.71	0.345	1.16	18.08	3.27	19.07	0.662

SD = standard deviation; FAs = Fixed appliance; CAs = Clear aligner, changes in TMJ parameters (T0 minus T1) for FAs and CAs within the non-extraction and extraction groups were analyzed using independent t-tests. *p ≤ 0.05 indicates statistical significance. Positive values indicate a decrease, and negative values indicate an increase

**Fig. 5:**
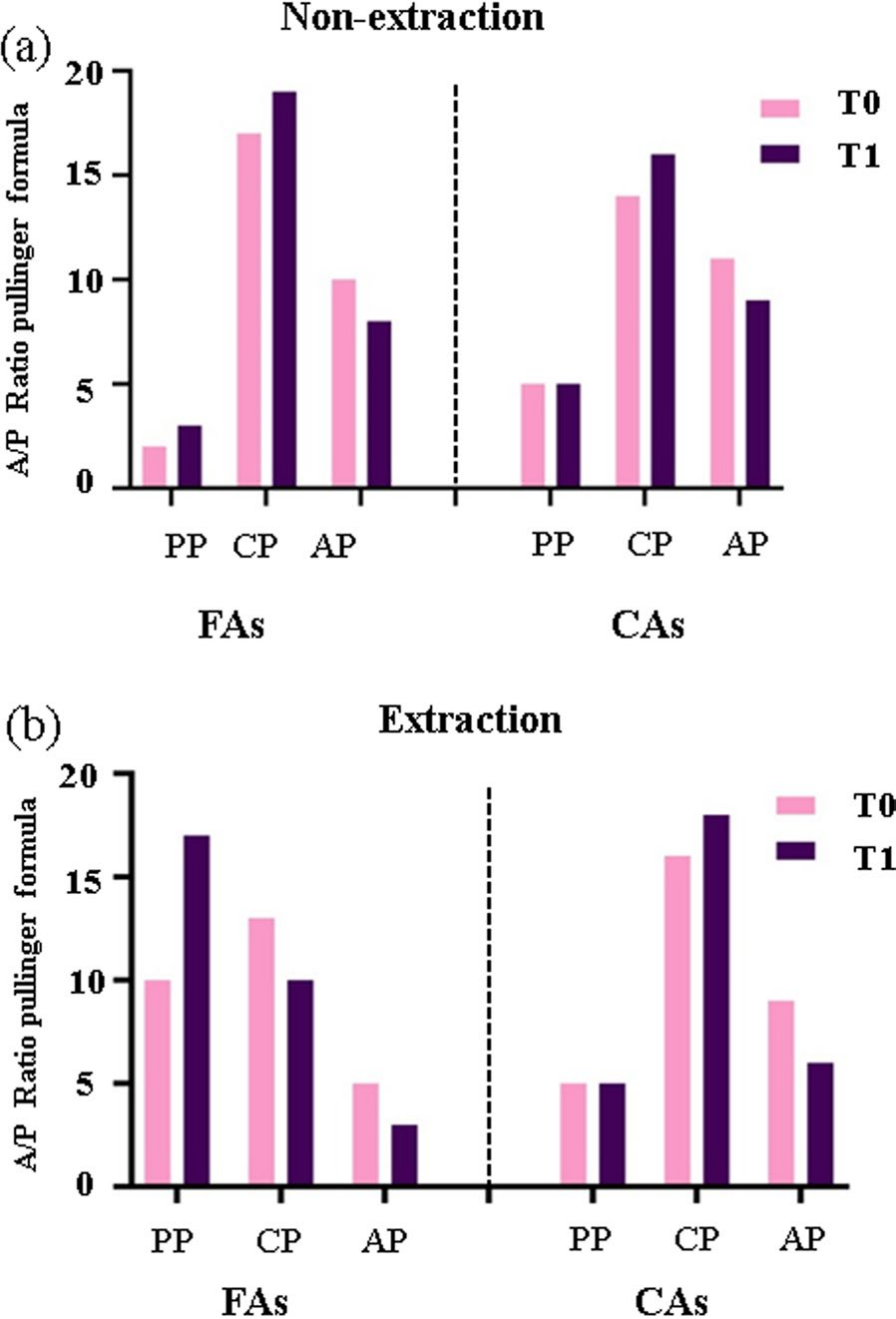
Changes in the anteroposterior ratio of condyle positioning for FAs and CAs within non-extraction and extraction treatment groups, calculated using the Pullinger formula, depicted as frequencies. **a** Shows the distribution of condylar positions—PP (posterior position), CP (centric position), and AP (anterior position)—for the non-extraction groups, while **b** presents the same data for the extraction groups

Figure 5 illustrates changes in the anteroposterior ratio of condyle positioning across treatment groups, as calculated using the Pullinger formula. In the non-extraction group, FAs experienced a slight increase in posterior position (PP), a rise in centric position (CP), and a decrease in anterior position (AP). On the other hand, CAs saw stable PP, increased CP, and reduced AP. The extraction group revealed more distinct changes: FAs showed a significant increase in PP, a decrease in CP, and a reduction in AP; CAs had stable PP, increased CP, and decreased AP. Overall, the data suggest a general increase in CP and a decrease in AP across both groups, with a notable increase in PP, particularly in FAs of the extraction group.

## Discussion

TMJ pretreatment values can assess changes and evaluate treatment outcomes after orthodontic or orthognathic procedures in adults. Detailed 3D measurements of TMJ structures help understand morphological and/or pathological alterations [25]. The strong correlation between intra- and inter-observer reliability confirms the precision of CBCT in identifying landmarks, making it superior for evaluating osseous structures in the TMJ region, unmatched by conventional methods [32]. This study is the first to use CBCT to evaluate TMJ changes in 3D before and after FAs and CAs, with and without premolars extractions, in adults with skeletal Class I malocclusion. The results offer valuable guidance for planning orthodontic treatment in patients without symptoms or those with mild TMD.

The study's sample selection criteria standardized variables influencing outcomes. Participants, all with skelelal Class I malocclusion, followed the same treatment protocol using either non-extraction or four first premolar extractions protocols. The same orthodontic appliances (FAs and CAs) and mechanics were used. This strict focus ensured comparability in initial severity and minimized bias. Both groups had almost identical baseline characteristics and underwent treatment for the same duration.

### TMJ comparisons in non-extraction group

In our study, both FAs and CAs groups, with almost similar baseline characteristics and treatment durations, use non-extraction methods to address moderate crowding. This involved distalization of posterior teeth and expansion of the dental arches to maintain a Class I relationship. Frequently, this required the extraction of upper third molars to allow the required and adequate movement of the first and second molars [36].

Our study showed that FAs significantly impact TMJ dynamics, with notable increases in the anteroposterior condylar position and decreases in the vertical condylar position and inclination. This suggests TMJ adaptation due to changes in occlusal forces and masticatory muscle activity following the orthodontic treatment. The increase in medial condylar inclination indicates condylar remodeling in response to the altered mechanical environment by FAs. These findings highlight the importance of monitoring TMJ health during and after orthodontic treatment to manage TMDs and ensure stable outcomes, emphasizing the biomechanical effects of orthodontic appliances on TMJ dynamics. Conversely, no significant changes were observed in patients treated with CAs, affirming their efficacy in maintaining TMJ measured values.

Our findings align with the hypothesis that the maxillary incisors can constrain the mandible, pushing it posteriorly due to the maxillary dentoalveolar complex's inability to move anteriorly [37]. Proper evaluation and adjustment of the sagittal positions of the first molars are essential for maintaining optimal vertical dimension and functional occlusion [38]. Additionally, muscle activity can influence condylar positioning, with lighter muscle contractions potentially positioning the condyle more inferiorly [39]. The neuromuscular system may adjust the condyle position downward in response to occlusal forces to optimize occlusal contact [40]. However, previous research indicates that the condylar position remains stable during orthodontic treatment, with no significant differences between extraction and non-extraction conditions [41]. These findings underscore the complex interplay between dental positioning, muscle activity, and condylar positioning in achieving optimal orthodontic outcomes.

The study examined condylar position changes using two methods: basal craniofacial reference planes and the Pullinger formula [34]. The Pullinger formula revealed a shift towards a more centric condylar position in both FAs and CAs groups post-treatment, indicating adaptive TMJ repositioning. Both groups showed an increase in the centric position and a decrease in the anterior position over time. The posterior position (PP) increased slightly in FAs but remained constant in CAs. The first method showed no statistical differences post-treatment in CAs cases. Changes in the condylar position determined by the Pullinger formula were also reflected using basal reference planes (MSP, HP, and VP), supporting the adaptive repositioning theory of the TMJ after orthodontic treatment [42].

The findings showed no significant differences in TMJ spaces and volumetric joint space in the CAs group. The correction in the CAs group is due to aligner technology, which enables precise 3D tooth movement by encircling the tooth crown and applying correction forces. This technology allows simultaneous correction of both the maxilla and mandible, enhancing treatment efficiency and stability during the maintenance stage [43].

### TMJ comparisons in extraction group

In Class I malocclusions, premolars are extracted to address tooth and arch length discrepancies and reduce anterior teeth protrusion. The extraction space is used to alleviate crowding and retract anterior teeth while preserving the position of the posterior teeth through effective anchorage, which typically does not change the vertical dimension [44].

Our findings indicated a significant increase in vertical dimension in the FAs group. Some argue that premolar extractions can decrease the vertical dimension of occlusion, leading to overclosure of the mandible, muscle foreshortening, and potentially resulting in TMDs [30]. However, our results challenge this perspective, demonstrating that vertical dimension can increase despite extractions, possibly due to molar extrusion and other contributing factors.

Our findings indicated significant changes in TMJ parameters in the FAs group, including a notable increase in the anteroposterior condylar position and an increase in the anterior joint space, while the posterior joint space decreased. Furthermore, the anteroposterior condylar joint position exhibited significant posterior shifts. These alterations may result from condylar rotation due to changes in the vertical dimension of occlusion and remodeling of the articular surfaces. TMJ can adaptively remodel, with similar condyle displacements observed during static clenching [45]. Orthodontic treatment triggers neuromuscular and skeletal adaptations, with dysfunction occurring only if changes surpass the patient’s adaptive threshold. Studies by Wyatt et al. [46] and Ali et al. [47] observed that maxillary anterior teeth retraction and premolar extractions can influence condyle position, making it more concentric post-treatment. Carlton and Nanda [42] found that premolar extractions significantly altered anterior and posterior joint spaces, particularly in Class II, division 1 malocclusion cases.

FAs significantly impacted the anteroposterior condylar position more than CAs, increasing anterior joint space and decreasing posterior joint space. These findings highlight the need to consider TMJ parameters in orthodontic planning to avoid potential TMDs. The study suggests that CAs mitigate common side effects of other orthodontic appliances and offer better control over TMJ parameters due to their design, encompassing teeth on all surfaces and applying appropriate forces through digitally planned attachments [48]. The anteroposterior shifts and increased vertical dimensions highlight the need for precise control during treatment to prevent maladaptive outcomes. These findings reinforce the importance of ongoing TMJ assessment during and after orthodontic treatment to possibly reduce risks of developing TMDs, especially in extraction cases. CAs are a favorable choice for patients prioritizing TMJ health, especially with joint sensitivities. Individualized appliance selection is crucial, and future research should examine whether TMJ remodeling enhances functionality or risks dysfunction.

This study provides insights into TMJ adaptations following orthodontic treatment but has certain limitations. The retrospective design limits control over variables such as patient compliance and precludes direct assessment of occlusal force distribution, which may have influenced the findings. To minimize such biases, future studies should consider randomized controlled trials or match patients based on their preferences to more accurately analyze the effects of different treatment modalities, free from external biases. Additionally, incorporating patient-reported outcomes, such as comfort, satisfaction, and quality of life, alongside clinical outcomes, would provide a more holistic understanding of treatment impacts. The focus on adult patients with Class I malocclusion restricts generalizability of the findings to other populations, such as adolescents or individuals with different malocclusions. Furthermore, the lack of long-term follow-up prevents evaluation of the stability of TMJ changes and their potential impact on the development of TMD risk. Future studies should incorporate diverse patient populations, longitudinal follow-up to assess the stability and progression of TMJ changes, and advanced imaging modalities such as magnetic resonance imaging (MRI) to assess soft tissue adaptations, including disc positioning and joint lubrication dynamics. Including occlusal and functional parameters, such as mastication patterns or occlusal force distribution would also enhance the understanding of TMJ responses to orthodontic treatment.

## Conclusion

Within the constraints of this study, the following conclusions were reached:

- Compared to CAs, FAs exhibited better control of the vertical dimension. The FAs group had significantly greater clockwise mandibular rotation than the CAs group. FAs, particularly in extraction cases, appeared to influence condylar positions and joint spaces more significantly.
- CAs were effective correction of skeletal Class I malocclusion with minimal impact on TMJ parameters, suggesting their suitability for cases where TMJ stability is a priority.
- These findings highlight the importance of tailoring orthodontic appliance selection to individual patient needs and TMJ health. Specifically, the results may inform the appliance choice based on the unique characteristics of each malocclusion. Regular monitoring TMJ parameters throughout treatment remains essential to optimize outcomes and minimize the risk of developing TMDs.

## Supplementary Information

Additional file 1.

## Data Availability

All data is available in the Orthodontics Department of the Hospital of Stomatology of China Medical University. The datasets of the current study are available from the corresponding author upon any request.
