# Supplementary material for: Three-dimensional quantitative temporomandibular joint changes in skeletal class I malocclusion treated with extraction and non-extraction protocols: a comparative study of fixed orthodontic appliances and clear aligners
Source: Prog Orthod. 2025 Jan 20;26:4. doi: 10.1186/s40510-024-00551-1 (PMC11743406; doi:10.1186/s40510-024-00551-1)
Supplement: Supplementary file 1 — Additional file 1. [file 40510_2024_551_MOESM1_ESM.docx]

| Supplementary Material 1 Definitions of the 3D TMJ landmarks used in the study | | |
| --- | --- | --- |
| **Landmark** | **Abb.** | **Definition** |
| Skeletal landmarks | | |
| Nasion^a^ | (N) | The juncture of the frontal nasal suture located at the most posterior curvature point on the nose bridge. |
| Sella^a^ | (S) | The central point of the hypophyseal fossa as identified through inspection. |
| Basion^a^ | (Ba) | The front-lower edge of the foramen magnum along the skull's midline base. |
| Incisive foramen^a^ | (IF) | The center of the incisive foramen is behind the central incisors on the midline of the maxillary palate. |
| Orbitale^b^ | (Or) | The lowest point on the bottom edge of both the right and left bony eye sockets. |
| Subspinale ^a^ | （A) | The most posterior concave point at the middle of the frontal maxilla |
| Supramental^a^ | （B） | The most posterior concave point at the middle mandibular symphysis process |
| Anterior nasal spine | (ANS) | The most forward projection at the lower art of nasal cavity, located midline of the maxilla. |
| Posterior nasal spine | (PNS) | The most pointed bony projection at the posterior nasal cavity on the midline of the palatine bone. |
| Porion^b^ | (Po) | The central point on the upper edge of the external auditory meatus on both the right and left sides. |
| Condylion^b^ | (Co) | The uppermost and rearmost point on the condyle's curvature. |
| Subspinale^a^ | （A） | The furthest back point on the maxilla curve between the anterior nasal spine and supradentale. |
| Submenale^a^ | （B） | The furthest back point on the mandibular curve between Infradentale and Pogonion. |
| Gnathion | （Gn） | The lowest and most forward point on the midline of the mandibular symphysis. |
| Gonnion^b^ | （Go） | The most posterior-inferior point on the mandibular gonial angle. |
| Sagittal View | | |
| Anterior condylar point^b^ | （ACP） | The most forward prominent point on the condylar head. |
| Posterior condylar point^b^ | （PCP） | The most rearward prominent point on the condylar head. |
| Superior condylar point^b^ | （SCP） | The highest point on the condylar head. |
| Articular tubercle^b^ | （AT） | The lowest point on the anterior tubercle. |
| Inferior meatus^b^ | （IM） | The lowest point on the external auditory meatus. |
| Anterior fossa^b^ | （AF） | The most anterior and inferior point on the inner anterior wall of the glenoid fossa. |
| Posterior fossa^b^ | （PF） | The most posterior and inferior point on the inner posterior wall of the glenoid fossa, aligned with the IM. |
| Anterior condyle neck point^b^ | （ANP） | The deepest point on the anterior concave wall of the condylar neck. |
| Posterior condyle neck point^b^ | （PNP） | The deepest point on the posterior concave wall of the condylar neck, approximately in line with the anterior neck point (ANP). |
| Anterior joint space  ”mandibular fossa point” ^b^ | （AJSf） | The most prominently posterior point of the inner anterior wall of the glenoid fossa, located closest to the anterior joint space condylar point (AJSc). |
| Anterior joint space  ”condylar point” ^b^ | （AJSc） | The most prominent anterior point of the condyle head, located closest to the anterior joint space fossa point（AJSf）. |
| Posterior joint space  ”mandibular fossa point” ^b^ | （PJSf） | The most prominent anterior point of the posterior inner wall of the glenoid fossa, opposite the nearest posterior condyle-fossa distance. |
| Posterior joint space  ”condylar point” ^b^ | （PJSc） | The most prominent posterior point of the condyle head, opposite the nearest posterior condyle-fossa distance. |
| Axial View |  |  |
| Lateral condylar point^b^ | （LCP） | The outermost point on the condylar head. |
| Condyle width “anterior point” ^b^ | （CWa） | The most forward prominent point of the condyle head in the widest region. |
| Condyle width “posterior point” ^b^ | （CWp） | The most rearward prominent point of the condyle head in the widest region. |
| Coronal view |  |  |
| Medial joint space “fossa point” ^b^ | （MJSf） | The outermost point of the medial wall of the mandibular fossa. |
| Medial condylar point^b^ | （MCP） | The innermost point on the condylar head. |
| Soft tissue mandibular fossa^b^ | （SMF） | The central and highest point of the soft tissue within the mandibular fossa. |
| Bony mandibular fossa^b^ | （BMF） | The central and highest point of the bony section within the mandibular fossa. |
| -^a^ unilateral point, ^b^ bilateral points.  -All points are identified using the clearest view in three dimensions, adjusted separately by the slice locator on each of the three planes. | | |

| Supplementary Material 2 Definitions of 3D TMJ reference lines and planes | | |
| --- | --- | --- |
| Name | **Abb.** | **Definition** |
| **Reference Lines** |  |  |
| Tuberculo-meatal line | (TM) | The line connecting AT and IM points. |
| Anteroposterior condylar line | (ACP-PCP) | The line between ACP and PCP. |
| Mediolateral condylar line | (MCP-LCP) | The line connecting MCP and MCP. |
| Sella-nasion line | (SN) | The line connecting by two-point S, N. |
| **Reference Planes** | | |
| Horizontal plane | (HP) | Constructed by three points: the right Or and the Po points on both sides. |
| Midsagittal plane | (MSP) | Constructed by three points: N, Ba, and IF. |
| Vertical Plane | (VP) | Constructed S and perpendicular on MSP and HP. |
| Mandibular fossa horizontal plane | (MFHP) | The plane tangent to the BMF point and parallel HP. |
| Mandibular plane | (MP) | Constructed using three points: the Gn and the Go points on both sides. |

| Supplementary Material 3 Definitions of 3D TMJ measurements used in the study | | |
| --- | --- | --- |
| **Measurement** | **Abb.** | **Definition** |
| Jaw relation measurements(°) |  |  |
| Sagittal jaw relation | ANB | The angle formed between 3‑points; A, N, and B. |
| Vertical jaw relation | MP-SN | The angle between SN line and MP. |
| Mandibular fossa dimension (mm) | | |
| Mandibular fossa height | MFH | Distance extends perpendicularly between BMF and TM line |
| Mandibular fossa width | MFW | Distance extends horizontally between AF and PF |
| Condylar dimension (mm) | | |
| Condylar length | CL | The mediolateral distance from MCP to LCP |
| Condylar width | CW | The anteroposterior condylar width CWa to CWp |
| Condylar position (mm) | | |
| Vertical condylar position | VCP | Distance extends vertically from SCP to HP |
| Anteroposterior condylar position | APCP | Distance extends anteroposterior from ACP to VP |
| Mediolateral condylar position | MLCP | Distance extends mediolaterally from MCP to MSP |
| Anteroposterior condylar joint position | APCJP | The anteroposterior condylar position inside the joint according to the formula of Pullinger. |
| Condylar inclination (°) | | |
| Mediolateral condylar inclination | MCI | Angle between ACP-PCP line and HP |
| Vertical condylar inclination | VCI | Angle between MCP-LCP line and VP plane |
| Anteroposterior condylar inclination | APCI | Angle between MCP-LCP line and MSP |
| TMJ spaces (mm) | | |
| Anterior joint space | AJS | Closest distance between AJSc and AJSf |
| Posterior joint space | PJS | Closest distance between PJSc and PJSf |
| Superior joint space | SJS | Closest distance between SCP and SMF |
| Medial joint space | MJS | Closest distance between MCP and MJSf |
| Volumetric total joint space (mm^3^) | VTJS | Total volumetric mandibular joint spaces (superior, anterior, and posterior) which enclosed by TM line |
| Note: (°) = degrees, mm = millimeters, mm² = square millimeters, mm³ = cubic millimeters. | | |

| Supplementary Material 4 Reliability analysis of the 3D TMJ measurements used in the study | | | | | | | | | |
| --- | --- | --- | --- | --- | --- | --- | --- | --- | --- |
|  | **Intra-observer reliability** | | | | **Inter-observer reliability** | | | |  |
| **Measurement** | **ICC** | **TEM** | **rTEM** | **R*** | **ICC** | **TEM** | **rTEM** | **R*** |  |
| MFH | 0.987 | 0.193 | 0.320 | 0.973 | 0.980 | 0.227 | 0.444 | 0.962 |  |
| MFW | 0.991 | 0.240 | 0.348 | 0.980 | 0.988 | 0.269 | 0.439 | 0.974 |  |
| CL | 0.995 | 0.222 | 0.271 | 0.989 | 0.993 | 0.242 | 0.324 | 0.987 |  |
| CW | 0.992 | 0.085 | 0.097 | 0.983 | 0.988 | 0.100 | 0.134 | 0.977 |  |
| APCP | 0.994 | 0.237 | 0.848 | 0.988 | 0.994 | 0.272 | 1.116 | 0.984 |  |
| VCP | 0.994 | 0.188 | 1.819 | 0.987 | 0.994 | 0.202 | 2.047 | 0.984 |  |
| MLCP | 0.994 | 0.214 | 0.105 | 0.988 | 0.988 | 0.319 | 0.234 | 0.975 |  |
| APCJP | 1.000 | 0.605 | 12.915 | 0.999 | 1.000 | 0.617 | 13.388 | 0.999 |  |
| ACPI | 0.994 | 0.671 | 0.629 | 0.989 | 0.994 | 0.090 | 0.312 | 0.979 |  |
| VCI | 0.993 | 1.143 | 2.290 | 0.986 | 0.992 | 1.177 | 2.427 | 0.986 |  |
| MCI | 0.994 | 0.460 | 2.738 | 0.989 | 0.994 | 0.477 | 2.949 | 0.988 |  |
| AJS | 0.991 | 0.077 | 0.223 | 0.980 | 0.988 | 0.084 | 0.267 | 0.976 |  |
| SJS | 0.991 | 0.068 | 0.135 | 0.981 | 0.986 | 0.081 | 0.194 | 0.972 |  |
| PJS | 0.993 | 0.066 | 0.166 | 0.987 | 0.990 | 0.078 | 0.235 | 0.981 |  |
| MJS | 0.990 | 0.088 | 0.296 | 0.980 | 0.990 | 0.748 | 0.785 | 0.987 |  |
| TMJ V | 0.983 | 8.724 | 27.158 | 0.968 | 0.983 | 8.871 | 28.128 | 0.966 |  |
| -ICC: Intra-class correlation coefficient TEM and rTEM indicate an absolute and relative technical error of measurement. | | | | | | | | | |
